# *Plasmodium falciparum* cytoadherence to ICAM-1 is associated with cerebral malaria

**DOI:** 10.1186/1475-2875-9-S2-P27

**Published:** 2010-10-20

**Authors:** Lucy B Ochola, Bethsheba R Siddondo, Harold Ocholla, Siana Nyka, Eva N Kimani, Thomas N Williams, Johnstone O Makale, Anne Liljander, Britta C Urban, Pete Bull, Tadge Szestak, Kevin Marsh, Alister G Craig

**Affiliations:** 1 KEMRI/Wellcome Trust Research Programme, P. O. Box 230, 80108 Kilifi, Kenya; 2 Liverpool School of Tropical Medicine, Pembroke Place, Liverpool L3 5QA, UK; 3 Dar es Salaam University College of Education, Faculty of Science, P.O. Box 2329, Dares Salaam, Tanzania; 4 Department of Medicine Solna, Karolinska Institutet, Stockholm, Sweden

## 

The pathology of sever malaria is in part related to the pro-inflammatory nature of the host response but a number of other factors are also thought to be involved, including the interaction between infected erythrocytes and endothelium. This phenotype involves a range of host receptors and the parasite-derived variant antigen PfEMP1, which is expressed on the surface of the infected erythrocyte membrane. Previous studies have suggested a role for ICAM-1 in the pathology of cerebral malaria, although these were inconclusive. In this study we measured the binding to CD36 and ICAM-1 of patient isolates from varying clinical syndromes under static and flow conditions. We also used mutant ICAM-1 proteins to characterise the key contact residues on ICAM-1 and produce a detailed binding phenotype. Our results show that increased binding to CD36 is associated with uncomplicated malaria while ICAM-1 adhesion under flow conditions is raised in parasites from cerebral malaria cases. The pattern of ICAM-1 binding has also been investigated using mutant ICAM-1 proteins and indicates that isolates from severe malaria are biased towards a binding signature also seen with ITO4, a laboratory isolate selected for binding on human endothelium with similar receptor expression to that seen in the brain.

